# Short Communication: The Peripheral Cannabinoid CB_1_ Receptor Antagonist AM6545 Modifies Cardiovascular Effects of Endocannabinoids in DOCA-Salt Rats

**DOI:** 10.3390/ijms27104449

**Published:** 2026-05-15

**Authors:** Patryk Remiszewski, Eberhard Schlicker, Emilia Grzęda, Jolanta Weresa, Marek Toczek, Barbara Malinowska

**Affiliations:** 1 Department of Experimental Physiology and Pathophysiology, Medical University of Bialystok, 15-222 Bialystok, Poland; patryk.remiszewski@umb.edu.pl (P.R.); emilia.grzeda@up.poznan.pl (E.G.); jolanta.weresa@umb.edu.pl (J.W.); marek.toczek@umb.edu.pl (M.T.); 2 Department of Pharmacology and Toxicology, University of Bonn, 53127 Bonn, Germany; e.schlicker@uni-bonn.de; 3 Department of Animal Physiology and Biochemistry, Poznan University of Life Sciences, 60-637 Poznan, Poland

**Keywords:** AM6545, cannabinoid receptors, DOCA-salt, endocannabinoids, methanandamide, URB597

## Abstract

Peripherally restricted (‘second-generation’) cannabinoid CB_1_ receptor (CB_1_R) antagonists have been suggested to have therapeutic potential in numerous diseases. However, their effects on the cardiovascular system require further research. The peripheral CB_1_R antagonist AM6545 failed to modify the decrease in blood pressure (BP) elicited by inhibition of anandamide degradation in spontaneously hypertensive rats. The aims of the present study were to examine the effect of AM6545 on BP and its interaction with endocannabinoid-evoked effects in deoxycorticosterone acetate (DOCA)-salt rats. For this purpose, we applied methanandamide (MethAEA), a stable analogue of anandamide, and URB597, an inhibitor of its degradation, in urethane-anesthetized animals. AM6545 did not affect BP by itself. MethAEA elicited a biphasic effect (a rise in BP, followed by its fall); both phases were antagonized by AM6545. URB597 induced a monophasic hypotensive effect, which was abolished by AM6545 in DOCA-salt rats but further enhanced in control animals. AM6545 also unmasked an additional increase in BP after URB597 in both groups of rats. In conclusion, AM6545 modifies the cardiovascular effects of endocannabinoids in hypertension in a model-dependent manner. The cardiovascular effects of CB_1_R antagonists should be carefully evaluated when assessing their potential therapeutic significance, as they may unmask an increase in BP.

## 1. Introduction

Cannabinoid CB_1_ receptor (CB_1_R) antagonists suggest therapeutic potential for the treatment of metabolic diseases, such as obesity, diabetes, and dyslipidemia, as well as fibrotic disorders of the liver, heart, kidney, skin, and lung [1,2,3,4,5,6,7]. The brain penetrant (‘first-generation’) CB_1_R antagonist rimonabant was used over a period of a few years for the treatment of obesity and metabolic syndrome but had to be withdrawn from clinical use because of its unwanted neuropsychiatric side effects [1]. Peripherally restricted (‘second-generation’) CB_1_R antagonists have shown promising results in preclinical models of obesity and metabolic syndrome, alcoholic and non-alcoholic liver steatosis, liver and pulmonary fibrosis, and renal diseases [1,4,5,6,7,8]. The peripheral CB_1_R antagonist monlunabant (MRI-1891) proved to be efficacious and safe in a randomised, placebo-controlled, phase 2a clinical trial in adults with obesity and metabolic syndrome [9].

(Endo)cannabinoids like anandamide (AEA) are the endogenous agonists of cannabinoid receptors including CB_1_Rs and exert complex cardiovascular effects that are more pronounced in hypertension. Interestingly, both brief pressor and prolonged depressor effects are at least partially dependent on central and peripheral cannabinoid CB_1_ receptors [5,10,11,12,13]. The final direction of cardiovascular effects of (endo)cannabinoids is dependent on the model of hypertension [5,10,11].

Thus, in hypertensive (mRen2)27 rats, acute and chronic oral administration of rimonabant decreased blood pressure (BP) [14]. By contrast, intravenous (i.v.) injection of rimonabant and/or of another brain-penetrant CB_1_R antagonist, AM251, increased BP and/or heart rate (HR) (1) in anesthetized and conscious spontaneously hypertensive (SHR) rats [15,16,17], (2) in rats in which hypertension was induced by angiotensin II (Ang II), or (3) in salt-sensitive Dahl rats kept on 8% NaCl [15]. Moreover, in SHR, brain-penetrant CB_1_R antagonists enhanced the brief pressor response to AEA and inhibited the prolonged hypotension evoked by AEA or inhibitors of fatty acid amide hydrolase (FAAH) that degrade AEA (URB597 or AM3506) [15,16,18].

On the other hand, the peripheral CB_1_R antagonist AM6545 failed to diminish BP evoked by AM3506 in SHR [16] and in rats with metabolic syndrome-induced hypertension [19] but reversed the fall in BP induced by the non-selective CB_1_R agonist CP55940 (given i.v.) into a marked increase in normotensive rats [12].

The deoxycorticosterone acetate (DOCA)-salt model of secondary hypertension is a low renin and volume-overloaded form of hypertension connected with a salt-rich diet, one of the main lifestyle factors leading to hypertension [20,21]. Interestingly, there are marked differences in the hypertension-elicited changes in the endocannabinoid system between SHR and DOCA-salt hypertension. Thus, in comparison to normotensive controls the following was found: (1) the plasma and cardiac levels of the two major endocannabinoids, AEA and 2-arachidonoylglycerol (2-AG), were lower in SHR but higher in DOCA-salt rats [22]; (2) the function of presynaptic inhibitory CB_1_Rs on sympathetic nerve fibers innervating resistance vessels was unchanged in SHR but enhanced in DOCA-salt rats [22,23]; (3) the expression of CB_1_Rs in the cardiac left ventricle was unchanged in SHR but decreased in DOCA-salt [10]; (4) chronic administration of URB597 diminished BP and HR in DOCA-salt rats but not in SHR [24].

The aims of the present study were as follows: (1) to examine the effect of the peripheral cannabinoid CB_1_ receptor antagonist AM6545 on BP in DOCA-salt rats; (2) to study its influence on the cardiovascular effects elicited by the stable AEA analogue methanandamide (MethAEA) and by URB597, an inhibitor of AEA degradation.

## 2. Results

### 2.1. General

Basal systolic (SBP) blood pressure (mmHg), diastolic (DBP) blood pressure (mmHg), and heart rate (HR) (beats/min) before administration of MethAEA 0.3 µmol/kg or of URB597 3 µmol/kg were 106 ± 9, 63 ± 9, and 354 ± 23, respectively, in urethane-anesthetized normotensive rats (*n* = 11) and 101 ± 6, 57 ± 6, and 369 ± 15, respectively, in DOCA-salt hypertensive animals (*n* = 10). The peripheral CB_1_R antagonist AM6545 15 µmol/kg given intraperitoneally (i.p.) 45 min before anesthesia did not modify basal cardiovascular parameters significantly. Thus, the respective basal SBP, DBP, and HR values were 112 ± 8, 58 ± 7, and 371 ± 16 in normotensive rats (*n* = 14) and 114 ± 5, 63 ± 4, and 380 ± 16 in DOCA-salt rats (*n* = 9).

### 2.2. AM6545 Reduces Pressor and Depressor Effects of MethAEA

**Figure 1:**
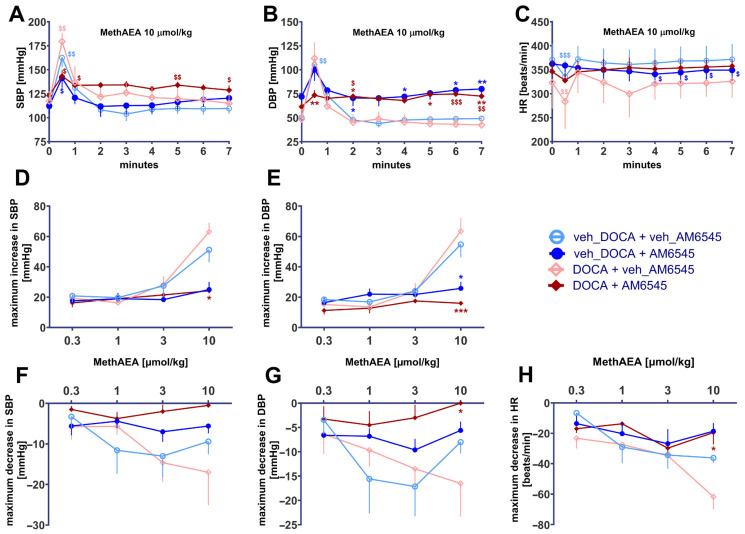
Influence of AM6545 on systolic-, diastolic blood pressure (SBP, DBP) and heart rate (HR) induced by methanandamide (MethAEA) in urethane-anesthetized normotensive (veh_DOCA) and DOCA-salt hypertensive rats (DOCA). AM6545 (15 μmol/kg, i.p) or its vehicle (veh) was administered 90 min before the first dose of MethAEA. Four increasing doses of MethAEA were administered i.v. with sufficient time for recovery to the preinjection value. (**A**–**C**) time dependence; (**D**,**E**) maximal increases and (**F**–**H**) maximal decreases in SBP, DBP, and/or HR. Means ± SEM of *n* = 4–9 animals. ^$^ *p* < 0.05, ^$$^ *p* < 0.01, ^$$$^ *p* < 0.001 in comparison to time 0; * *p* < 0.05, ** *p* < 0.01, *** *p* < 0.001 in comparison to the respective values without AM6545.

Intravenous administration of MethAEA produced biphasic cardiovascular effects both in normotensive and DOCA-salt rats. Thus, brief pronounced pressor effects (increases in SBP and DBP lasting for about 1 min) were followed by prolonged but weaker hypotension (Figure 1A,B). As shown in Figure 1D,E, MethAEA 0.3–3 µmol/kg increased SBP and DBP by about 20 mmHg whereas its highest dose (10 µmol/kg) caused increases by about 60 mmHg; the effects were comparable in normotensive and DOCA-salt rats. The maximal MethAEA-induced decreases in SBP and DBP in normotensive and DOCA-salt rats were about 15 mmHg and occurred at 3 µmol/kg (normotensive rats) and 10 µmol/kg (DOCA-salt rats); the dose-response curve of MethAEA in normotensive rats was steeper than that in DOCA-salt rats (Figure 1F,G).

AM6545 attenuated the pressor response (SBP and DBP) to MethAEA in normotensive and DOCA-salt animals to a comparable extent (Figure 1D,E). By contrast, the effect of AM6545 on the depressor effect of MethAEA was much more marked in DOCA-salt than in normotensive rats. The depressor response to the highest dose of MethAEA (10 µmol/kg) was hardly affected by AM6545 in normotensive animals but was even abolished in DOCA-salt rats (Figure 1F,G).

**Figure 2:**
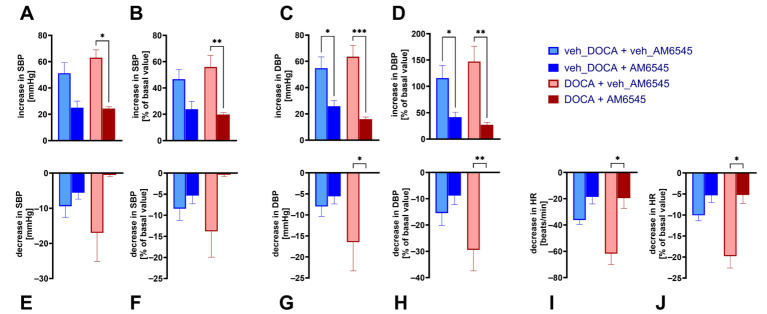
Impact of basal values on the influence of AM6545 on systolic-, diastolic blood pressure (SBP, DBP) and heart rate (HR) induced by methanandamide (MethAEA) 10 μmol/kg in urethane-anesthetized normotensive (veh_DOCA) and DOCA-salt hypertensive rats (DOCA). AM6545 (15 μmol/kg, i.p) or its vehicle (veh) was administered 90 min before the first dose of MethAEA. Four increasing doses of MethAEA (0.3, 1, 3, and 10 μmol/kg, see Figure 1) were administered i.v. with sufficient time for recovery to the preinjection value. (**A**–**D**) maximal increases and (**E**–**J**) maximal decreases in SBP, DBP, and/or HR. Results are expressed in absolute terms (left pair of columns in each panel) or as percent of the corresponding baseline level (right pair of columns). Means ± SEM of *n* = 4–5 rats. * *p* < 0.05, ** *p* < 0.01, *** *p* < 0.001 in comparison to the respective values without AM6545.

Decreases and increases in BP are dependent on basal values. To exclude the possibility that the impact of AM6545 on the cardiovascular effects of MethAEA was dependent on differences in basal parameters, we calculated the maximal increases and decreases obtained with MethAEA 10 µmol/kg in percentage of basal values. Figure 2 presents a comparison of absolute and normalized values (left vs. right pair of columns in each panel). Regardless of the type of calculation, the increase in SBP and DBP elicited by MethAEA 10 µmol/kg was strongly inhibited by AM6545 both in normotensive and DOCA-salt rats (Figure 2A–D); the antagonistic effect was more pronounced in DOCA-salt than in normotensive rats. With respect to the depressor effect of MethAEA 10 µmol/kg (and regardless of the type of calculation) the antagonistic effect of AM6545 was more marked in DOCA-salt than in normotensive rats (Figure 2E–H). The hypotensive effect of MethAEA was abolished by AM6545 in DOCA-salt rats but tended to be attenuated in their normotensive counterparts only (Figure 2E–H).

In contrast to the distinct changes in BP, MethAEA produced much less pronounced changes in HR. No consistent increase in HR was obtained (Figure 1C). However, MethAEA 10 µmol/kg decreased HR; the effect was more pronounced in DOCA-salt than in normotensive animals (Figure 1H and Figure 2I,J). The antagonistic effect of AM6545 was also more marked in DOCA-salt rats and reached significance in this group only.

### 2.3. AM6545 Unmasks Pressor Effect of URB597 in Normotensive and DOCA-Salt Rats but Decreases Its Depressor Influence in DOCA-Salt Rats Only

**Figure 3:**
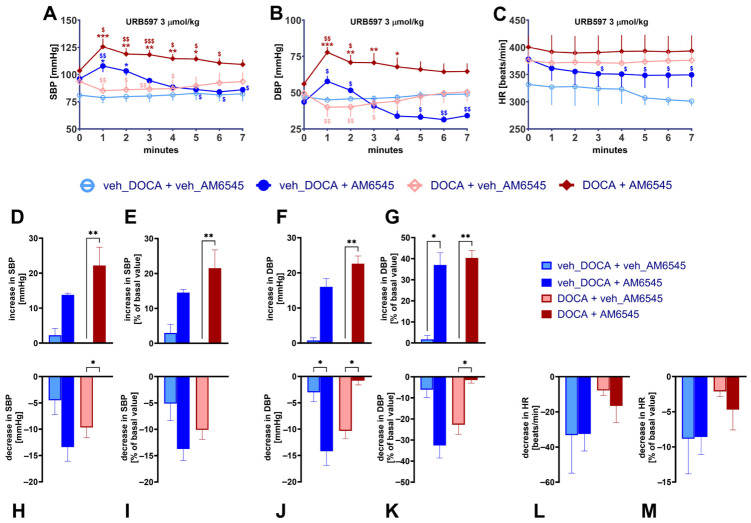
Influence of AM6545 on systolic-, diastolic blood pressure (SBP, DBP) and heart rate (HR) induced by URB597 in urethane-anesthetized normotensive (veh_DOCA) and DOCA-salt hypertensive rats (DOCA). AM6545 (15 μmol/kg, i.p) or its vehicle (veh) was administered 90 min before URB597 (3 μmol/kg, i.v.). (**A**–**C**) time dependence; (**D**–**G**) maximal increases and (**H**–**M**) maximal decreases in SBP, DBP, and/or HR. In each panel, values are expressed in absolute terms (left pair of columns) and as percent of baseline level (right pair of columns). Means ± SEM of *n* = 4–6 rats. ^$^ *p* < 0.05, ^$$^ *p* < 0.01, ^$$$^ *p* < 0.001 in comparison to time 0; * *p* < 0.05, ** *p* < 0.01, *** *p* < 0.001 in comparison to the respective values without AM6545.

Intravenous injection of URB597 3 µmol/kg induced a prolonged weak hypotension, maximally by about 5 mmHg for SBP and DBP, both for normotensive and DOCA-salt rats (Figure 3A,B,H–K). Prior administration of AM6545 unmasked a pressor effect of URB597 (SBP and DBP increased by about 15 mmHg) and enhanced its prolonged hypotension to about 15 mmHg in normotensive animals (Figure 3D–K). In DOCA-salt rats, AM6545 also unmasked strong increases in SBP and DBP (to about 20 mmHg) but, unlike in normotensive animals, it completely blocked the fall in SBP and DBP. We did not observe significant differences between maximal URB597-induced increases and decreases in SBP and DBP between normotensive and DOCA-salt rats nor did we find any impact of basal values (Figure 3E,G,I,K vs. Figure 3D,F,H,J).

URB597 failed to increase HR (Figure 3C). Decreases were weak and one may see the following from Figure 3L,M: (1) the effect of URB597 was stronger in normotensive than in DOCA-salt rats; (2) the effect was not changed by AM6545 in normotensive but increased in DOCA-salt rats, although statistical significance was not reached.

## 3. Discussion

The aim of our study was to examine (1) the effect of the peripheral cannabinoid CB_1_ receptor antagonist AM6545 on blood pressure and (2) its interaction with endocannabinoids in DOCA-salt rats. For this purpose, we applied MethAEA, a stable analogue of the well-known endocannabinoid AEA, and URB597, an inhibitor of AEA degradation [25]. The peripherally restricted antagonist AM6545 shares a high affinity (K_i_ of 3.3 nM) and selectivity for the CB_1_R with rimonabant, which belongs to the first generation of CB_1_R antagonists. Unlike rimonabant, AM6545 displays a markedly reduced penetration into the brain; the brain/plasma concentration ratios following acute parenteral or oral administration and removal of intravascular fluids were 0.8 and 0.03, respectively. Importantly, in contrast to rimonabant, AM6545 did not antagonize the cannabinoid-induced catalepsy, hypomotility, and hypothermia, i.e., the prototypical effects based on central CB_1_Rs [26]. Experiments were performed in rats anesthetized with urethane since its influence on the tonic activity of the autonomic nervous system is less marked than that of other anesthetics [27], and it did not modify the pressor effect of AEA [28]. We examined only male (but not female) rats, since, to the best of our knowledge, cardiovascular effects of (endo)cannabinoids under in vivo conditions have been so far examined in male rodents only. We applied the model of the volume overload DOCA-salt hypertension, which due to its salt-rich diet resembles one of the main lifestyle factors leading to hypertension in humans and showed clear differences in the endocannabinoid system compared to SHR, a genetic rat model of hypertension (for details, see Section 1. Introduction).

We did not observe differences in the basal cardiovascular parameter between urethane-anesthetized DOCA-salt rats and sham-operated normotensive controls (similar to Bunag et al. [29]). This could be expected since an increase in sympathetic nerve activity plays a crucial role in the development of hypertension in the DOCA-salt model, and the reduction of the sympathetic tone by urethane significantly lowers resting BP and HR when compared to unanesthetized animals [20,27,30]. In our previous study, basal BP and HR in urethane-anesthetized DOCA-salt pithed rats were even lower than in their normotensive controls [23]. We expressed maximal changes in cardiovascular parameters both as absolute values and as percentages of the respective basal values to exclude the influence of interindividual differences in basal values on the final results; comparable results were obtained with both types of calculations.

MethAEA elicited a biphasic response of SBP and DBP, i.e., a short lasting brief pressor effect followed by a prolonged and weaker hypotension. The first phase of the classical triple response to AEA, i.e., a reflex fall in BP and HR (Bezold–Jarisch reflex; reviewed in Malinowska et al. [10]), was not observed since it occurs upon rapid injection of AEA or MethAEA only [31]. In the current study, we concentrated on the interplay between reflex-independent pressor and depressor effects and for this reason refrained from rapid administration of substances. As a consequence, we do not discuss the changes in HR, which are most distinct in phase I [31]. A slight prolonged fall in SBP and DBP was observed after injection of URB597, an inhibitor of AEA degradation. Like in other publications in which FAAH inhibitors (URB597 or AM6506) were examined, their i.v. injection elicited a monophasic effect only [15,16].

Both pressor (MethAEA) and depressor responses (MethAEA and URB597) only tended to be higher in DOCA-salt rats in comparison to their normotensive controls. By contrast, AEA, nanoformulated-AEA, URB597, and AM6506 decreased BP to a much higher extent in SHR and in Ang II-induced hypertension than in their respective normotensive controls [15,16,32]. Note that in the latter studies hypertensive rats had a much higher basal BP than their normotensive controls.

As in our previous paper [12] and in rats with metabolic syndrome-induced hypertension [19], AM6545 failed to modify basal cardiovascular parameters by itself. This is in marked contrast to studies in which CB_1_R antagonists of the first generation like rimonabant and/or AM251 decreased BP in (mRen2)27 rats [14] or increased it (1) in SHR rats [15,16,17], (2) rats rendered hypertensive by Ang II, or (3) salt-sensitive Dahl rats kept on 8% NaCl [15]. However, AM6545 completely blocked the fall in SBP and DBP elicited by MethAEA and URB597 in DOCA-salt rats. By contrast, AM6545 completely failed to modify the fall in BP induced by the FAAH inhibitor AM6506 in SHR [16]. This is another difference in the endocannabinoid system/effects between SHR and DOCA-salt hypertension (see Section 1. Introduction), indicating that the effect of AM6545 is dependent on the model of hypertension.

Cannabinoid CB_1_Rs are known for their vasodilatory effect [11,33]. We found that MethAEA-stimulated relaxation was enhanced in the resistance arteries of the mesenteric system isolated from DOCA-salt rats and expression of CB_1_Rs was upregulated in this vascular bed in comparison to their normotensive controls [33]. The above vasodilatory effects of MethAEA were reduced by AM6545 in DOCA-salt rats and by AM6545 and capsazepine (an antagonist of the Transient Receptor Potential Vanilloid type 1 (TRPV1)) in normotensive animals [33]. The higher expression of CB_1_Rs in the resistance arteries of DOCA-salt rats might explain that the peripheral CB_1_R antagonist totally blocked the fall in BP induced by MethAEA and URB597 in DOCA-salt rats.

In their normotensive counterparts, also other receptors are involved in the hypotensive response to MethAEA; e.g., the so called non-CB_1_ cannabinoid vascular receptors (identical to the orphan receptor GPR18 [34]), which are sensitive to O-1918, may come into play [31]. Interesting enough, the hypotensive effect of URB597 (unlike that of MethAEA) was even increased or tended to be increased by AM6545. URB597 is known to potentiate the relaxation of rat isolated small mesenteric arteries to AEA (but not to Meth-AEA) [35], which is mediated mainly by TRPV1 receptors [33]. One should also keep in mind that AEA, but not MethAEA, might act via vasorelaxant or vasoconstrictor metabolic products [36].

AM6545 reduced or tended to reduce the pressor effect of MethAEA both in normotensive and hypertensive rats. We previously showed the involvement of central CB_1_Rs, β_2_-adrenergic, NMDA, and thromboxane A_2_ receptors in the pressor effect of AEA and MethAEA [12,37]. Although AM6545 is a peripherally restricted CB_1_R antagonist, its acute administration enhanced mouse memory performance through a central and peripheral noradrenergic mechanism [38]. Moreover, subchronic i.p. administration of AM6545 enhanced cognitive performance and induced hippocampal synaptic plasticity changes in mice [39]. In addition, peripherally-restricted pharmacological inhibition of CB_1_R reduces ethanol drinking in mice [40,41] and food intake in mice with diet-induced obesity [42,43]. One cannot exclude that a similar crosstalk between peripheral tissues and the brain took place in the current study.

In contrast to MethAEA, the pressor response to URB597 was noticed only after previous blockade of peripheral CB_1_Rs. Similarly, AM6545 reversed the hypotensive effect of CP55940 in urethane-anesthetized rats into a centrally mediated hypertensive response [12]. How can we explain that the pressor response of URB597 was unmasked by AM6545? (1) It might result from the blockade of presynaptic inhibitory CB_1_Rs on sympathetic nerve endings innervating the resistance vessels, the function of which has been shown to be enhanced in DOCA-salt rats [33]. Accordingly, another CB_1_R antagonist AM251 enhanced the neurogenic vasopressor response by itself in DOCA-salt rats [23]. (2) Since the increase in BP also occurred in control rats, other mechanisms might come into play. Thus, vasoconstrictors like Ang II and thromboxane A_2_, in addition to their contractile action, also stimulate the rapid biosynthesis of endocannabinoids that restrict agonist-induced contraction in systemic or pulmonary arteries acting via CB_1_Rs. Accordingly, CB_1_R antagonists/inverse agonists may enhance the action of vasoconstrictors blocking the negative feedback built up by endocannabinoids [44].

Importantly, our short communication has some limitations. Thus, different results may be obtained if one were to use any of the following: (1) another model of experimental hypertension (see Section 1. Introduction); (2) another type of anesthesia; (3) conscious animals (different responses to AEA or MetAEA were noticed in anesthetized vs. conscious rats [10]); (4) female rats. So far, the sex dependence of cardiovascular effects of (endo)cannabinoids has not been studied under in vivo conditions. In vitro, the vasodilator response to AEA in mesenteric arteries isolated from SHR was diminished in hypertensive males but not modified in female rats [45]. Moreover, the addition of AM6545 to human coronary arteries pretreated with capsaicin caused a more pronounced inhibition of vasorelaxation in females than males [46]. It would be interesting to examine the influence of AM6545 on the CB_1_R-dependent hypotension and bradycardia induced by the stable analog of 2-AG, 2-AG-ether [47]. As shown in a very recent publication on hypertensive patients, levels of 2-AG tended to be higher in comparison to normotension whereas the AEA concentration did not show any differences [48].

In conclusion, the peripherally restricted CB_1_R antagonist AM6545 does not affect BP in DOCA-salt rats. The stable endocannabinoid analogue MethAEA elicited a biphasic BP effect (rise in BP, followed by fall) in our study; both phases were antagonized by AM6545. The inhibitor of AEA degradation, URB597, elicited a monophasic hypotensive effect, which was abolished by AM6545 in DOCA-salt rats but further increased in control rats. AM6545 also unmasked an additional increase in BP in both groups of rats. It is of interest that (1) the differences in cardiovascular effects of AM6545 between DOCA-salt rats and their normotensive counterparts occurred, although the baseline parameters had an identical level, and (2) the influence of AM6545 in hypertension is dependent on the hypertension model. Since AM6545 was able to unmask an endocannabinoid-evoked BP increase, one should check the cardiovascular effects of CB_1_R antagonists carefully when studying their potential therapeutic significance.

## 4. Materials and Methods

### 4.1. Animals

Forty-four male Wistar rats were used in the current experiments. All surgical procedures and experimental protocols adhered to the ARRIVE guidelines, European Directive 2010/63/EU, and to Polish regulations, while approval from the local Animal Ethics Committee in Białystok, Poland was also received. The study was carried out in compliance with the Three Rs Principle (reduction, replacement, and refinement). Rats were obtained from the Center of Experimental Medicine of the Medical University of Białystok (Poland); they were housed under a 12 h light/12 h dark cycle and had unrestricted access to food pellets and water.

### 4.2. DOCA-Salt Hypertension

Rats with an initial weight of approximately 130–200 g were anesthetized with pentobarbitone sodium (i.p., 70 mg/kg, i.e., 300 μmol/kg). The right kidney was removed in all rats via a right lateral abdominal incision. After a 1-week recovery period, hypertension was induced by deoxycorticosterone acetate (DOCA, subcutaneously, s.c.) at a dose of 25 mg/kg (i.e., 67 μmol/kg; 0.4 mL/kg) twice weekly for 4 weeks. Simultaneously, their drinking water was replaced with a 1% NaCl solution. Sham operated rats (controls) received the vehicle for DOCA twice weekly and drank tap water. Four weeks after the first dose of DOCA or its vehicle, systolic blood pressure (SBP) and heart rate (HR) were measured in conscious rats using the non-invasive tail-cuff method with the Non-Invasive Blood Pressure Controller (Hugo Sachs Elektronik-Harvard Apparatus, March-Hugstetten, Germany). Only DOCA-salt rats with a SBP higher than 150 mmHg were considered as hypertensive.

### 4.3. Experimental Protocol

**Figure 4:**
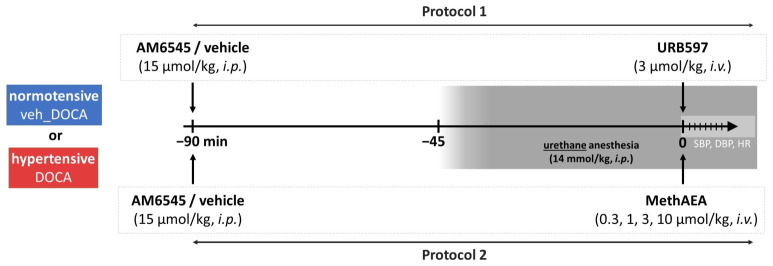
Experimental design used to examine cardiovascular responses to URB597 and methanandamide (MethAEA) in deoxycorticosterone acetate hypertensive rats (DOCA)-salt and their normotensive controls (veh_DOCA), with or without peripheral CB_1_ receptor blockade using AM6545. Conscious animals were injected with AM6545 (15 μmol/kg, i.p.) or its vehicle. After 45 min, urethane anesthesia (14 mmol/kg, i.p.) was induced and another 45 min later URB597 (3 μmol/kg, i.v.; Protocol 1) or the lowest of four increasing doses of MethAEA (0.3, 1, 3, 10 μmol/kg, i.v.; Protocol 2) was administered; the next higher dose of MethAEA was given as soon as the cardiovascular effects to the previous dose had returned to baseline levels. Baseline values of systolic- (SBP) and diastolic (DBP) blood pressure, as well as heart rate (HR), were recorded before administration of each compound or dose. The numbers on the axis indicate time intervals (in minutes).

The experimental protocol is shown in Figure 4. Four weeks after unilateral nephrectomy, hypertensive and normotensive rats (now weighing 280–350 g) were anesthetized with urethane (14 mmol/kg = 1250 mg/kg) i.p. The trachea was cannulated. Systolic and diastolic blood pressure (SBP and DBP, respectively) was measured from the right carotid artery connected with a transducer (ISOTEC; Hugo Sachs Elektronik-Harvard Apparatus GmbH, March-Hugstetten, Germany) via polyurethane catheters filled with heparinized saline (100 units/mL) to prevent blood clot formation. Heart rate (HR) was recorded from the electrocardiogram (ECG) through subcutaneous electrodes. Body temperature was maintained constant at approximately 37 °C using a heating pad (Bio-Sys-Tech, Białystok, Poland) and monitored by a rectal probe transducer (Physitemp BAT10; Physitemp Instruments, Inc., Clifton, NJ, USA). The left femoral vein was cannulated for i.v. injection of drugs administered in a volume of 0.5 mL/kg. After surgical procedures, cardiovascular parameters were allowed to stabilize. About 20 min later, experiments were performed.

The peripheral CB_1_R antagonist, AM6545 (15 μmol/kg = 8.35 mg/kg) or its vehicle, was administered i.p. to both groups of rats 45 min before the onset of anesthesia and 90 min before MethAEA or URB597 [12]. In one series of experiments, four increasing doses of the stable AEA analogue methanandamide (MethAEA; 0.3, 1, 3, and 10 µmol/kg, i.e., 0.109, 0.362, 1.085, and 3.616 mg/kg, respectively) were administered i.v. with sufficient time for recovery to the preinjection value. The first dose of MethAEA was given 90 min after AM6545 or its vehicle. In an additional series of experiments, the AEA degradation inhibitor URB597 (3 µmol/kg = 1.02 mg/kg) or its vehicle was injected i.v. 90 min after the administration of AM6545 or its vehicle. The assignment of animals to the above protocols, groups, and cage location as well as the order of the treatment and measurements were random. No a priori exclusion criteria were established. All investigators were aware of the animal allocation during experiments and the statistical analysis.

### 4.4. Drugs

Drugs were obtained from the following sources: AM6545 (5-(4-[4-cyanobut-1-ynyl]phenyl)-1-(2,4-dichlorophenyl)-4-methyl-*N*-(1,1-dioxo-thiomorpholino)-1*H*-pyrazole-3-carboxamide) from Sigma-Aldrich (St. Louis, MO, USA); DOCA (deoxycorticosterone acetate) from Sigma-Aldrich (Steinheim, Germany); R-(-)-methanandamide from Tocris Cookson (Bristol, UK); URB597 ([3-(3-carbamoylphenyl)phenyl] N-cyclohexylcarbamate) from Cayman Chemical Company (Ann Arbor, MI, USA); urethane (ethyl carbamate) from Sigma (Munich, Germany); pentobarbitone sodium from Biowet (Puławy, Poland). Drugs were dissolved in saline with the following exceptions: AM6545 was dissolved in dimethyl sulfoxide (DMSO) using gentle warming before dilution with Tween 80 and saline (4% DMSO, 1% Tween 80, 95% saline) for i.p. administration. Methanandamide was supplied as a 10 mg/mL emulsion in soya oil/water (1:4) by the manufacturer. URB597 was dissolved in a mixture of DMSO and Tween 80 (1:2) and then diluted in saline (3:7) immediately before the experiment.

### 4.5. Data Analysis

The individual rat was considered the experimental unit within the studies. Results are presented as means ± SEM. The sample size was estimated on the basis of our previous experiments in this model, the available literature, and the guidelines by Curtis et al. [49]. No a priori sample size calculation was done. Due to a few cases of failure in measurement of hemodynamic parameters the number of results in each group is not uniform. To quantify the effects of the peripheral CB_1_ receptor antagonist AM6545 on MethAEA- and URB597-induced changes in cardiovascular parameters, data are expressed either as absolute values (in mmHg or beats/min) or as values normalized to the basal level measured at time 0 and presented as a percentage of basal (% basal). The parameters “maximum increase” and “maximum decrease” refer to the greatest increase or the greatest decrease observed during the 7 min experimental period, expressed relative to the basal value (time 0).

Normality of data distribution was assessed using the Shapiro–Wilk test. For comparisons of mean values, one-way analysis of variance (ANOVA) was applied when data were normally distributed, whereas the Kruskal–Wallis test was used when the assumption of normality was not met. If a significant overall effect was detected, post hoc analysis was performed using Šídák’s multiple comparisons test following ANOVA or Dunn’s multiple comparisons test following the Kruskal–Wallis test. For comparisons between time points, paired tests were used: the paired Student’s t-test for normally distributed data or the Wilcoxon signed-rank test when normality was not assumed. Differences were considered statistically significant at *p* < 0.05.

## Data Availability

Data generated or analyzed during this study are available from the corresponding author upon reasonable request.

## Abbreviations

The following abbreviations are used in this manuscript:

2-AG	2-Arachidonoylglycerol
AEA	Anandamide
Ang II	Angiotensin II
ANOVA	Analysis of variance
BP	Blood pressure
CB_1_R	Cannabinoid receptor type 1
DBP	Diastolic blood pressure
DMSO	Dimethyl sulfoxide
DOCA	Deoxycorticosterone acetate
FAAH	Fatty acid amide hydrolase
GPR18	G-protein-coupled receptor 18
HR	Heart rate
i.p.	Intraperitoneal administration
i.v.	Intravenous administration
K_i_	Inhibitory constant
MethAEA	Methanandamide
NMDA	N-methyl-D-aspartate
SBP	Systolic blood pressure
s.c.	Subcutaneous administration
SEM	Standard error of the mean
SHR	Spontaneously hypertensive rats
TRPV1	Transient receptor potential vanilloid type 1
veh	Vehicle
